# Correlation of HbA1c Level with Lipid Profile in Type 2 Diabetes Mellitus Patients Visiting a Primary Healthcare Center in Jeddah City, Saudi Arabia: A Retrospective Cross-Sectional Study

**DOI:** 10.3390/diseases11040154

**Published:** 2023-10-31

**Authors:** Abdulaziz Yahya Sharahili, Shabir Ahmad Mir, Sahar ALDosari, Md Dilshad Manzar, Bader Alshehri, Ayoub Al Othaim, Fayez Alghofaili, Yahya Madkhali, Kamal Shaker Albenasy, Jazi S. Alotaibi

**Affiliations:** 1 Department of Medical Laboratory Sciences, College of Applied Medical Science, Majmaah University, Al Majmaah 11952, Saudi Arabia; 421103846@s.mu.edu.sa (A.Y.S.); s.aldosari@mu.edu.sa (S.A.); b.alshehri@mu.edu.sa (B.A.); ay.alothaim@mu.edu.sa (A.A.O.); f.alghofaily@mu.edu.sa (F.A.); y.madkhali@mu.edu.sa (Y.M.); k.albenasy@mu.edu.sa (K.S.A.); 2 Health and Basic Sciences Research Center, Majmaah University, Al Majmaah 11952, Saudi Arabia; 3 Department of Nursing, College of Applied Medical Science, Majmaah University, Al Majmaah 11952, Saudi Arabia; m.manzar@mu.edu.sa (M.D.M.); jalotaibi@mu.edu.sa (J.S.A.)

**Keywords:** diabetes mellitus, dyslipidemia, lipid profile, HbA1C, Saudi Arabia

## Abstract

Introduction: Type 2 diabetes mellitus (T2DM) patients are at high risk of dyslipidemia, which in turn is associated with macrovascular diseases, such as heart diseases and stroke, and microvascular diseases, such as neuropathy and nephropathy. There are contradictory findings in the literature regarding the relationship between glycated hemoglobin (HbA1c) and the lipid profile among T2DM patients. This study was performed to investigate the association between HbA1c level and the lipid profile in elderly T2DM patients at a primary care hospital in Jeddah City, Saudi Arabia. Methods: This study is a retrospective cross-sectional study conducted at the Prince Abdul Majeed Healthcare Center (PAMHC) in Jeddah, Saudi Arabia. The sociodemographic and clinical data of the T2DM patients who had visited the PAMHC from 1 January 2020 to 31 December 2021, were collected from the data registry of the PAMHC and analyzed for publication. Results: The study included a total of 988 T2DM patients (53.3% male). Of the participants, 42.9% were aged between 55 and 64 years. Dyslipidemia parameters were presented as high LDL-c (in 60.3% cases), low HDL-c (in 39.8% cases), high triglycerides (in 34.9% cases), and high total cholesterol (in 34.8% cases). The correlation of HbA1c with total cholesterol (TC) and triglycerides (TGs) was positively significant, thereby highlighting the important link between glycemic control and dyslipidemia. A mean increase of 4.88 mg/dL and 3.33 mmHg in TG level and diastolic blood pressure, respectively, was associated with the male gender, in comparison to the female gender. However, the male gender was significantly associated with the reduction in the mean cholesterol level, BMI, HbA1c, HDL-c, and LDL-c by 11.49 mg/dL, 1.39 kg/m^2^, 0.31%, 7.47 mg/dL, and 5.6 mg/dL, respectively, in comparison to the female gender. Conclusions: The results of this study show that HbA1c was significantly associated with cholesterol and triglyceride levels in the T2DM patients included in the study. Our findings highlight the important relationship between glycemic control and dyslipidemia.

## 1. Introduction

The prevalence of diabetes mellitus in Saudi Arabia has dramatically increased from 3.4% in 1996 to more than 20% in the recent years, which is majorly attributed to changes in lifestyle [1,2]. Saudi Arabia ranked seventh among the top ten countries in regard to diabetic mellitus prevalence [2]. The complications associated with type 2 diabetes mellitus (T2DM) increase the burden of disease globally due to prolonged morbidity. About 366 million people have developed diabetes in 2011, and 552 million are expected to be diabetic in 2030 [3,4]. It is estimated that 380 million people have type 2 diabetes and about 400 million have impaired glucose tolerance. There are many cases that remain undiagnosed with diabetes, so it is underestimated [5,6].

It is estimated that about 7 million of the Saudi population are diabetic and almost about 3 million are pre-diabetics [7]. The spread of sedentary lifestyles and the adoption of Western dietary habits, high in refined carbohydrates and fat, are driving an increase in the number of people with obesity-related diabetes [8]. Diabetes, the most common non-communicable disease in Saudi Arabia, is having an increasing impact on rates of morbidity, risk of hypertension, atherosclerosis, and dyslipidemia [7].

According to the American Diabetes Association (ADA), a glycated hemoglobin (HbA1c) level ≥6.5% is recommended for diagnosis of diabetes, while pre-diabetic patients could be diagnosed with HbA1c levels in the range of 5.7% to 6.4%. Reasons supporting the use of HbA1c level in the diagnosis and monitoring of diabetes mellitus are small intra-individual variability, a reflection of the average plasma glucose for the previous 2–3 months, in addition to the feasibility of the assessment without the need for fasting [9]. However, the use of HbA1c is taken with caution due to lower test sensitivity in certain patient groups, such as those with sickle cell anemia, or in certain populations, such as the Asian population [10]. 

Diabetic patients are at high risk of developing dyslipidemia (atherogenic dyslipidemia), which is associated with macrovascular diseases, such as heart diseases and stroke, and microvascular diseases, such as neuropathy and nephropathy [11,12]. Atherogenic dyslipidemia is characterized by high triglyceride (TG) levels, low high-density lipoprotein (HDL) levels, and high low-density lipoprotein (LDL) levels in serum [13]. Some studies suggested that HbA1c could be used as a reliable predictor of dyslipidemia and heart disease [14,15]. Despite the use of HbA1c as an indicator of glycemic control and associated diabetes complications, some studies doubt the association between HbA1c and dyslipidemia [16,17,18,19,20]. Among Indian diabetic patients, no significant association was found between HbA1c and the lipid profile [21]. Additionally, some studies found a negative association between HbA1c and low-density lipoprotein (LDL) [22], while others found a positive relationship between HbA1c and triglycerides [12,23]. Only triglyceride was significantly associated with HbA1c in a study conducted in 206 diabetic patients in Saudi Arabia [24]. These contradicting findings highlighted the need for further investigations of the association between HbA1c and the lipid profile among diabetic patients. Hence, this study was performed to investigate the association between HbA1c and the lipid profile in a relatively large sample of patients with T2DM.

## 2. Materials and Methods

### 2.1. Study Site and Design

This cross-sectional retrospective study was conducted at the Prince Abdul Majeed Healthcare Center (PAMHC) in Jeddah City, Saudi Arabia. The PAMHC is a specialized health center that provides emergency and routine healthcare services for the surrounding population. The sociodemographic and clinical data of the newly diagnosed type 2 diabetes mellitus (T2DM) patients were collected retrospectively from the medical records of the PAMHC by using the random sampling technique. The data of the diabetic patients were retrospectively collected for a period of 2 years (from 1 January 2020 to 31 December 2021).

### 2.2. Inclusion Criteria

Elderly patients (≥45 years old) with recent diagnosis of T2DM, based on the American Diabetes Association (ADA) criteria [9,25], were included in this study. Accordingly, patients were considered to have T2DM if they fulfilled one of the following criteria: “HbA1c ≥ 6.5%, Fasting Plasma Glucose (FPG) ≥ 126 mg/dL (7.0 mmol/L), 2 h postprandial plasma glucose ≥ 200 mg/dL (11.1 mmol/L) during an oral glucose tolerance test (OGTT), or random plasma glucose ≥ 200 mg/dL (11.1 mmol/L)” [9].

### 2.3. Exclusion Criteria

Patients who were taking lipid-lowering therapy or those with cardiovascular diseases, endocrinal conditions, liver function impairment, or renal problems were excluded from the study. Furthermore, patients with mental problems were also excluded from the study.

### 2.4. Sample Size

The sample size was calculated based on a previous study [24] with the odds ratio (r) = 0.16, beta error = 0.20, and alpha error = 0.05. The minimal sample size was found to be 304 according to the method described in the book of Hulley, 2007 [26]. Our study included 988 T2DM patients, indicating the adequateness of the sample size in this study.

### 2.5. Study Variables

The lipid profile of the diabetic patients, including total cholesterol (TC), high-density lipoprotein-cholesterol (HDL-c), low-density lipoprotein-cholesterol (LDL-c), and triglycerides (TG), represent the dependent variables of our study, whereas the independent variables included the HbA1c levels of the diabetic patients. The other additional characteristics of the patients, including age, educational level, occupation, marital status, blood pressure, and body mass index (BMI), were characterized as the confounding variables for this study.

### 2.6. Statistical Analysis

The data was entered and analyzed by the Statistical Package of Social Science SPSS, version 26. Descriptive statistics, such as frequencies and percentages, were calculated to summarize nominal and ordinal data, whereas the mean, median, and standard deviation or range were calculated to describe numerical variables. The correlation coefficient was calculated for the targeted association. The t-test was used if the independent variable was dichotomized during analysis. The chi-squared test was used to evaluate the association between categorical determinants and the outcome variables. Regression analysis was used to estimate adjusted odds ratios. Any *p*-value < 0.05 was considered as an indication of a statistically significant association or difference. We performed several standard tests to ascertain that the dataset satisfied the multiple linear regression analysis requirement. Four separate regression models were run with continuous variables of cholesterol level (mg/dL), triglyceride level (mg/dL), HDL-c level (mg/dL), LDL-c level (mg/dL) as dependent variables (DVs), and gender, age, nationality, habits, marital status, occupation, education, BMI (kg/m^2^), systolic blood pressure, diastolic blood pressure, glucose level (mg/dL), and HBA1C (mg/dL) were independent variables (IVs). Some of the IVs were continuous variables, and some were categorical variables. The independence of observations was indicated by a Durbin–Watson statistic of 2.034, 1.94, 1.968, and 2.049. By fitting a straight line to the partial regression plots, a linear connection between the DV and all IVs was discovered. As evidenced by an approximately zero mean value and a standard deviation that was almost equal to 1, standardized residuals had a distribution that was almost normal. By manually examining the histogram for frequency versus standardized residuals plot for each of the four models, it was possible to confirm that the standardized residuals had a normal distribution. As evidenced by tolerance values more than 0.2 [27] and variance inflation factor (VIF) values less than 10 [28], the multicollinearity criteria were satisfied. Furthermore, there were no concerns about the IV–IV correlation coefficients as all values were less than 0.7 (Supplementary Table S1).

Upon analysis of the line of best fit in the plot of studentized residual vs. standardized projected values, all four models did not meet the homoscedasticity criterion. The heteroskedasticity was found in all four models, as evidenced by the F test for heteroskedasticity and the Breusch–Pagan test for heteroskedasticity. As homoscedasticity was violated in all four models, therefore, all four multiple linear regression models were run after adjustment. The heteroskedasticity-adjusted standard errors, *p*-values, and t-statistics were estimated using SPSS 28.0 version. After ascertaining for absence of data entry errors, regression analysis was performed without excluding highly influential/multivariate outlier cases because excluding extreme values solely due to their extremeness can distort the results by removing information about the variability inherent in the clinical samples. 

### 2.7. Ethical Approval

The collected data and the patient information were kept anonymous to assure the privacy of patients and were only used for research purposes. The study protocol was approved by the ethical research committee on Publication Ethics (Directorate of Health Affairs—Jeddah) under the ethical approval number A01346. Before participation, the aim, methods, and expected results of this study were described to the ethical approval committee.

## 3. Results

### 3.1. Socio-Demographic Characteristics of T2DM Patients

This study included sociodemographic and clinical data of 988 T2DM patients who visited the PAMHC in Jeddah from 1 January 2020 to 31 December 2021. The majority of the participating T2DM patients were male (53.3%) (Table 1). More than 2/5th of the T2DM patients (42.9%) belonged to the age group of 55 to 64 years. Most of the patients (86.3%) were non-smokers. More than 1/5th of the T2DM patients (20.6%) were living as single (unmarried/divorced/widowed) and about 1/3rd (34.5%) of the patients were employed (Table 1). Regarding the educational level, the majority of the patients (95.3%) were educated with 21.2% of patients being university graduates or post-graduates.

### 3.2. Biochemical Parameters of T2DM Patients

The BMI of the diabetic patients ranged from 17.40 to 83.30 with a mean of 30.8 ± 5.78. More than half of the patients (52.8%) were obese with different grades of obesity. The mean HbA1c level and fasting blood glucose level among the patients was 8.36 ± 1.77 and 185.48 ± 45.82, with 97.1% and 99.3% of the patients having abnormal HbA1c and fasting blood glucose levels, respectively. The mean TC level was 187.5 ± 47.41 mg/dL with 34.8% of the patients having abnormal levels of TC, while the mean TG level was 144.74 ± 81.11 mg/dL and more than one-third of the patients (34.9%) had abnormal TG levels. The mean levels of LDL and HDL were 114.28 ± 39.87 mg/dL and 44.42 ± 16.94 mg/dL, respectively, and the prevalence of their abnormal levels was 60.3% and 39.8%, respectively (Table 2).

Both the gender differed significantly with regards to BMI, t(986) = 3.792, *p* ≤ 0.001; diastolic blood pressure, t(986) = −5.41, *p* < 0.001; HBA1c, t(986) = 2.817, *p* = 0.005, Total Cholesterol, t(986) = 3.826, *p* < 0.001, HDL-c, t(986) = 7.085, *p* < 0.001, and LDL-c, t(986) = 2.207, *p* = 0.028 (Table 3).

### 3.3. Multiple Linear Regression Model—Associated Factors of Total Cholesterol Level

The increasing level of total cholesterol was associated with a lower BMI (b = −0.595, *p* = 0.025), higher diastolic blood pressure (b = 0.394, *p* = 0.02), higher glucose level (b = 0.107, *p* = 0.02), higher HbA1c level (b = 2.544, *p* = 0.04), being single (b = 8.330, *p* = 0.03), age group 45–54 years (b = 15.149, *p* = 0.043), age group 55–64 years (b = 12.708, *p* = 0.047), female sex (b = 10.439, *p* = 0.01), and tertiary education level (b = 19.984, *p* = 0.03) (model adjusted R^2^ = 0.081, *p* < 0.05) (Table 4).

### 3.4. Multiple Linear Regression Model—Associated Factors of Triglyceride Level

Increasing level of triglyceride was associated with higher diastolic blood pressure (b = 0.665, *p* = 0.035) and higher HbA1c level (b = 7.927, *p* < 0.001) (model adjusted R^2^ = 0.041, *p* < 0.05) (Table 5).

### 3.5. Multiple Linear Regression Model—Associated Factors of LDL-c Level

The increasing level of LDL-c was associated with age group 55–64 years (b = 10.246, *p* = 0.045) (model adjusted R^2^ = 0.036, *p* < 0.05) (Table 6). The other parameters/factors, including the HbA1c, did not show any significant correlation with the LDL-c level of the T2DM patients.

### 3.6. Multiple Linear Regression Model—Associated Factors of HDL-c Level

The increasing level of HDL-c was associated with higher diastolic blood pressure (b = 0.099, *p* = 0.043) and female sex (b = 6.658, *p* < 0.001) (model adjusted R^2^ = 0.048, *p* < 0.05) (Table 7). The other parameters/factors, including the HbA1c, did not show any significant correlation with the HDL level of the T2DM patients.

## 4. Discussion

The increased risk of cardiovascular disease (CVD) in T2DM patients is partly due to the abnormalities in the lipid profile accompanying T2DM. Various studies have reported the association between HbA1c and one or more parameters of the lipid profile in T2DM patients, and some studies suggested HbA1c as a possible biomarker for recognizing the abnormal lipid profile of T2DM patients and for identifying the T2DM patients at risk of CVD [24,29,30,31]. Our results show a significant positive correlation between HbA1c and triglycerides and between HbA1c and total cholesterol. These findings agree with some previous studies which also reported a significant positive correlation between HbA1c and one or more parameters of the lipid profile in T2DM patients [24,32,33]. Our results and the previous reports highlight the important link between glycemic control and dyslipidemia [24,31,33,34]. This indicates that HbA1c is directly associated with dyslipidemia in T2DM diabetic patients and indirectly helps in assessing the risk of micro- and macrovascular problems [12,35]. Insulin resistance is considered the cause of dyslipidemia in T2DM patients. An inadequate secretion or function of insulin is reported to be linked with increased TG levels in T2DM patients through several mechanisms [24,36]. However, the correlation between HbA1c and LDL-c in the present study was found to be weak-positive and statistically insignificant, and no correlation was observed between HbA1c and HDL-c. These results are consistent with some earlier studies which also reported no correlation between these parameters [21,24] and inconsistent with others [11,35]. The current study also showed that the older age of diabetic patients was significantly associated with total cholesterol level, which is in line with a similar previous study which also reported a positive significant association between LDL-c and age [37]. Additionally, our results showed that the diastolic blood pressure of T2DM patients was significantly and positively correlated with their blood cholesterol and blood triglyceride levels.

In the present study, 97.1% of the T2DM patients had abnormal HbA1c, and LDL-c was the most prevalent (60.3%) dyslipidemia parameter among the T2DM patients. These results are in line with some previous studies on dyslipidemia among diabetic patients [32,38]. However, compared to our results, several other studies have reported a lower prevalence of high LDL-c levels in diabetic patients [39,40,41,42,43]. The difference could be attributed to various factors, including regional differences, differences in study design, and selection of study population. This indicates that factors other than T2DM might be involved in the development of dyslipidemia.

The gender-wise comparison revealed that females had significantly higher values for LDL-c, HDL-c, HbA1c, BMI, total cholesterol, and diastolic blood pressure as compared to males. Few other studies have reported similar results [14,24,44]. However, there are some differences between our results and those of other studies [15,45]. Gender-related differences in lipid parameters may be due to sex hormone-dependent changes in body lipid distribution that result in alterations in lipoprotein levels [46]. Other factors that may contribute to the difference in results include BMIs and age, as well as time since diagnosis of T2DM. Our participants had a mean BMI > 30, indicating they were obese. The association of obesity and physical inactivity with poor blood sugar control has been reported earlier [47].

According to the World Health Organization, the global prevalence of hypercholesterolemia among adults is 39%, with the Eastern Mediterranean region ranking as the third most hypercholesterolemia prevalent region (38.4% of the adults having high levels of cholesterol) [48,49]. In Gulf states, the prevalence of hypercholesterolemia is commonly above 50% in the general population [49]. The prevalence of hypercholesterolemia in the T2DM patients included in this study was 34.8%, which is slightly lower than the average prevalence in the Gulf states. This could be attributed to the strict change in the diet and lifestyle that is usually associated with elderly diabetic patients. Some of the differences between our study and studies conducted in other countries regarding the association between HbA1c and lipid profile parameters may be due to the difference in population level, as dyslipidemia is already prevalent in Saudi Arabia, even among non-diabetics [50].

The main strength point of this study is the inclusion of a relatively large number of study subjects (sample size, *n* = 988 T2DM patients). Moreover, statistical power in this study was adequate to detect significant associations between parameters. However, the main limitation is the retrospective cross-sectional approach to study a time-variant event such as glycemic control and dyslipidemia. Future research should focus on a prospective approach and compare baseline values and the temporal change of HbA1c and the dyslipidemia profile. In addition, this study contains single region-based data from a single primary healthcare center, therefore, the results cannot be directly generalized to the general population of the region. 

## 5. Conclusions

In the present study, we report that the level of dyslipidemia among diabetic patients is high as more than half of the patients possessed high LDL-c levels and most of the patients had abnormal levels in at least one lipid parameter. The correlation between HbA1c and each of triglycerides and cholesterol was a positive significant correlation. These findings highlight the important relationship between glycemic control and dyslipidemia.

## Figures and Tables

**Table 1:** Sociodemographic characteristics of the study subjects (clinically diagnosed T2DM patients who attended the Prince Abdul Majeed Healthcare Center in Jeddah from January 2020 to December 2021).

Characteristics	Frequency	Percentage (%)
**Gender**		
Female	461	46.7
Male	527	53.3
**Age**		
45–54 years	289	29.3
55–64 years	424	42.9
65–74 years	190	19.2
75 years and above	85	8.6
**Smoking**		
Smoker	135	13.7
Non-Smoker	853	86.3
**Marital status**		
Single/Divorced/Widowed	204	20.6
Married	784	79.4
**Employment**		
Employed	341	34.5
Unemployed	393	39.8
Retired	254	25.7
**Educational level**		
Primary education	296	30.0
Secondary education	437	44.2
University or post-graduate education	209	21.2
Illiterate	46	4.7

**Table 2:** Distribution of the clinical/biochemical parameters among the clinically diagnosed T2DM patients who visited the Prince Abdul Majeed Healthcare Center in Jeddah from January 2020 to December 2021.

Clinical/Biochemical Parameters	Frequency (Percentage)	Minimum	Maximum	Mean	SD
BMI		17.40	83.30	30.84	5.78
BMI categories:					
Underweight	2 (0.2)				
Normal	147 (14.9)				
Overweight	318 (32.2)				
Obese grade I	301 (30.5)				
Obese grade II	159 (16.1)				
Obese grade III	61 (6.2)				
HbA1c		5.8	15.9	8.36	1.77
Normal	29 (2.9)				
High	959 (97.1)				
(normal = less than 6.5%)					
Fasting Blood Glucose		117	427.00	185.48	45.82
Normal	7 (0.7)				
High	981 (99.3)				
(Normal ≥ 126 mg/dL)					
Total Cholesterol		69.0	376.0	187.50	47.41
Normal	644 (65.2)				
High	344 (34.8)				
(normal = less than 200 mg/dL)					
Triglycerides		27.0	751.0	144.74	81.11
Normal	643 (65.1)				
High	345 (34.9)				
(normal = Less than 150 mg/dL)					
HDL-c		4.5	389.0	44.42	16.94
Normal	595 (39.8)				
Low	393 (39.8)				
(normal = greater than 40 mg/dL)					
LDL-c		9.2	296.0	114.28	39.87
Normal	392 (39.7)				
High	596 (60.3)				
normal = less than 100 mg/dL)					

**Table 3:** Gender-wise distribution of the clinical/biochemical parameters among the clinically diagnosed T2DM patients who visited the Prince Abdul Majeed Healthcare Centre in Jeddah from January 2020 to December 2021.

Clinical/Biochemical Parameter	Gender	Mean	Std. Deviation	Minimum	Maximum	t-Statistics	*p*
BMI	Female	31.58	5.47	19.67	52.68	3.792	**<0.001**
	Male	30.19	5.97	17.40	83.30		
SysBP	Female	136.53	19.47	91.00	280.00	−1.423	0.155
	Male	138.23	18.11	98.00	209.00		
DiaBP	Female	70.47	9.74	47.00	114.00	−5.41	**<0.001**
	Male	73.80	9.56	47.00	100.00		
Glucose	Female	185.86	48.30	117.00	427.00	0.244	0.807
	Male	185.15	43.59	124.00	421.00		
HBA1C	Female	8.53	1.90	5.80	15.60	2.817	**0.005**
	Male	8.22	1.63	6.20	15.90		
Total Cholesterol	Female	193.63	48.04	69.00	353.00	3.826	**<0.001**
	Male	182.14	46.25	95.00	376.00		
Triglycerides	Female	142.14	72.75	30.00	604.00	−0.942	0.346
	Male	147.02	87.77	27.00	751.00		
HDL-c	Female	48.40	21.21	5.50	389.00	7.085	**<0.001**
	Male	40.93	10.89	4.50	148.00		
LDL-c	Female	117.26	39.48	9.20	233.60	2.207	**0.028**
	Male	111.66	40.07	25.40	296.00		

**Table 4:** Associated determinants/factors affecting the Total Cholesterol level in clinically diagnosed T2DM patients who attended PAMHC for diagnosis/follow-up/disease management from 1 January 2020 to 31 December 2021.

Independent Variable	Beta Coefficient	Robust Standard Error ^#^	T Values ^#^	*p*-Values ^#^	Model Unadjusted R^2^; Adjusted R^2^; *p*-Value
BMI	−0.595	0.265	−2.243	0.025	0.096; 0.081; <0.05
Systolic blood pressure	−0.035	0.085	−0.417	0.677	
Diastolic blood pressure	0.394	0.169	2.333	0.020	
Glucose	0.107	0.046	2.294	0.022	
HbA1C	2.544	1.240	2.051	0.040	
Marital Status Single/divorced	8.330	3.838	2.171	0.030	
Married	Ref				
Age (In years) 45–54	15.149	7.470	2.028	0.043	
55–64	12.708	6.392	1.988	0.047	
65–74	9.683	6.408	1.511	0.131	
75 and above	Ref				
Gender Female	10.439	4.048	2.579	0.010	
Male	Ref				
Habits Smoker	−2.023	4.519	-0.448	0.654	
Non-smoker	Ref				
Occupation Employed	5.146	4.585	1.122	0.262	
Unemployed	5.610	4.876	1.151	0.250	
Retired	Ref				
Education Primary education	7.257	7.768	0.934	0.350	
Secondary education	10.308	8.320	1.239	0.216	
Tertiary education	19.984	9.197	2.173	0.030	
Illiteracy	Ref				
Intercept	108.132	17.903	6.040	0.000	

^#^ Heteroskedasticity adjusted standard error—a robust estimator of the covariance matrix of the parameter estimates with a jackknife estimator.

**Table 5:** Associated determinants/factors affecting the triglyceride level in clinically diagnosed T2DM patients who attended the PAMHC for diagnosis/follow-up/disease management from 1 January 2020 to 31 December 2021.

Independent Variable	Beta Coefficient	Robust Standard Error ^#^	T Values ^#^	*p*-Values ^#^	Model Unadjusted R^2^; Adjusted R^2^; *p*-Value
BMI	−0.255	0.414	−0.617	0.537	0.057; 0.041
Systolic blood pressure	−0.137	0.148	−0.922	0.357	
Diastolic blood pressure	0.665	0.315	2.114	0.035	
Glucose	0.060	0.072	0.827	0.408	
HbA1C	7.927	1.995	3.974	0.000	
Marital Status Single/divorced	7.477	7.522	0.994	0.320	
Married	Ref				
Age (In years) 45–54	0.394	11.922	0.033	0.974	
55–64	14.020	10.345	1.355	0.176	
65–74	3.208	9.219	0.348	0.728	
75 and above	Ref				
Gender Female	−9.680	6.928	−1.397	0.163	
Male	Ref				
Habits Smoker	1.029	8.983	0.115	0.909	
Non-smoker	Ref				
Occupation Employed	8.554	7.920	1.080	0.280	
Unemployed	9.640	8.206	1.175	0.240	
Retired	Ref				
Education Primary education	−10.502	13.959	−0.752	0.452	
Secondary education	−7.030	15.030	−0.468	0.640	
Tertiary education	−3.610	16.408	−0.220	0.826	
Illiteracy	Ref				
Intercept	42.326	33.820	1.251	0.211	

^#^ Heteroskedasticity adjusted standard error—a robust estimator of the covariance matrix of the parameter estimates with a jackknife estimator.

**Table 6:** Associated determinants/factors affecting the LDL-c level in clinically diagnosed T2DM patients who attended the PAMHC for diagnosis/follow-up/disease management from 1 January 2020 to 31 December 2021.

Independent Variable	Beta Coefficient	Robust Standard Error ^#^	T Values ^#^	*p*-Values ^#^	Model Unadjusted R^2^; Adjusted R^2^; *p*-Value
BMI	−0.394	0.225	−1.750	0.080	0.052; 0.036
Systolic blood pressure	0.025	0.074	0.338	0.736	
Diastolic blood pressure	0.094	0.145	0.647	0.518	
Glucose	0.077	0.040	1.932	0.054	
HbA1C	1.510	1.011	1.493	0.136	
Marital Status Single/divorced	6.094	3.278	1.859	0.063	
Married	Ref				
Age (In years) 45–54	10.853	6.073	1.787	0.074	
55–64	10.246	5.093	2.012	0.045	
65–74	9.147	5.296	1.727	0.084	
75 and above	Ref				
Gender Female	4.522	3.385	1.336	0.182	
Male	Ref				
Habits Smoker	−5.131	3.830	−1.340	0.181	
Non-smoker	Ref				
Occupation Employed	4.981	4.142	1.202	0.229	
Unemployed	2.648	4.139	0.640	0.522	
Retired	Ref				
Education Primary education	−0.500	5.957	−0.084	0.933	
Secondary education	1.171	6.159	0.190	0.849	
Tertiary education	8.377	6.888	1.216	0.224	
Illiteracy	Ref				
Intercept	72.323	15.164	4.769	0.000	

^#^ Heteroskedasticity adjusted standard error—a robust estimator of the covariance matrix of the parameter estimates with a jackknife estimator.

**Table 7:** Associated determinants/factors affecting the HDL-c level in clinically diagnosed T2DM patients who attended the PAMHC for diagnosis/follow-up/disease management from 1 January 2020 to 31 December 2021.

Independent Variable	Beta Coefficient	Robust Standard Error ^#^	T Values ^#^	*p*-Values ^#^	Model Unadjusted R^2^; Adjusted R^2^; *p*-Value
BMI	−0.029	0.068	−0.424	0.672	0.063; 0.048
Systolic blood pressure	0.011	0.027	0.412	0.680	
Diastolic blood pressure	0.099	0.049	2.023	0.043	
Glucose	−0.021	0.014	−1.426	0.154	
HbA1C	0.462	0.381	1.212	0.226	
Marital Status Single/divorced	0.266	1.512	0.176	0.861	
Married	Ref				
Age (In years) 45–54	4.067	2.198	1.850	0.065	
55–64	1.744	1.653	1.055	0.292	
65–74	2.905	2.179	1.333	0.183	
75 and above	Ref				
Gender Female	6.658	1.343	4.956	<0.001	
Male	Ref				
Habits Smoker	−0.575	1.063	−0.541	0.588	
Non-smoker	Ref				
Occupation Employed	−1.241	1.217	−1.020	0.308	
Unemployed	1.077	1.587	0.678	0.498	
Retired	Ref				
Education Primary education	4.415	2.325	1.899	0.058	
Secondary education	2.830	2.314	1.223	0.222	
Tertiary education	3.179	2.403	1.323	0.186	
Illiteracy	Ref				
Intercept	27.732	5.110	5.427	<0.001	

^#^ Heteroskedasticity adjusted standard error—a robust estimator of the covariance matrix of the parameter estimates with a jackknife estimator.

## Data Availability

The raw data file is available on reasonable request from the corresponding author.

## Supplementary Materials

The following supporting information can be downloaded at: https://www.mdpi.com/article/10.3390/diseases11040154/s1, Table S1: Bivariate correlation coefficients.
