# Supplementary material for: Correlation of HbA1c Level with Lipid Profile in Type 2 Diabetes Mellitus Patients Visiting a Primary Healthcare Center in Jeddah City, Saudi Arabia: A Retrospective Cross-Sectional Study
Source: Diseases. 2023 Oct 31;11(4):154. doi: 10.3390/diseases11040154 (PMC10660465; doi:10.3390/diseases11040154)
Supplement: Supplementary file 1 [file diseases-11-00154-s001.zip › diseases-2636958-supplementary.pdf]

Supplementary Table S1. Bivariate correlation coefficients

	Gender	Age	Habits	Marital status	occupation	Education	Systolic BP	Diastolic BP	Glucose	HBA1C	Cholesterol	Triglycerides	HDL	LDL
Gender		.010	-.278**	.049	.057	.044	.051	.176**	.016	-.062	-.121**	.000	-.291**	-.078*
Age			.206**	-.234**	.542**	-.391**	.104**	-.144**	-.049	.011	-.160**	-.007	-.062*	-.114**
Habits				-.072*	.177**	-.076*	-.003	-.100**	-.113**	-.081*	.003	-.031	.076*	.020
Marital status					-.094**	.079*	-.010	.038	.075*	.060	-.023	.015	-.003	-.024
occupation						-.259**	.047	-.108**	-.079*	-.042	-.127**	-.030	-.027	-.097**
Education							-.047	.094**	-.008	-.005	.097**	.039	-.025	.090**
Systolic BP								.456**	.065*	.028	.014	.012	.024	.019
Diastolic BP									.082**	.047	.086**	.088**	.021	.042
Glucose										.629**	.179**	.146**	-.017	.137**
HBA1C											.181**	.197**	.034	.131**
Cholesterol												.362**	.200**	.890**
Triglycerides													-.101**	.097**
HDL														.119**
LDL														

* p <.05, **p<.01; Pearson's correlation test was used for between continuous variables (Systolic BP, Diastolic BP, Glucose, HBA1C, Cholesterol, Triglycerides, HDL, LDL), and Spearman test used for between categorical variables (Gender, Age, Habits, Marital status, occupation, and Education), and categorical v/s continuous variable correlation.

BP: blood pressure; HBA1C: Glycated Hemoglobin A, HDL: High density lipoprotein; LDL: low density lipoprotein
